# The “Sandwich” technique using a lateral locking plate and intramedullary reconstruction plate with bone cement for osteoporotic proximal humerus fractures: a case series

**DOI:** 10.3389/fsurg.2026.1745435

**Published:** 2026-10-07

**Authors:** Yuqiang Wang, Yang Lv, Ziyan Zhang, Xuedi Sun, Shijie Wei, Chengdong Piao

**Affiliations:** Department of Orthopedics, The Second Hospital of Jilin University, Changchun, China

**Keywords:** bone cement, locking plate, medial column support, osteoporosis, proximal humerus fracture, reconstruction plate, sandwich technique

## Abstract

**Introduction:**

Although lateral locking plate fixation is widely used for displaced proximal humerus fractures, fixation failure remains a major concern in elderly osteoporotic patients, particularly when medial column defects are present. Poor cancellous bone quality reduces screw purchase, while medial comminution and metaphyseal bone voids weaken the ability of the proximal humerus to resist varus and axial loading. Existing augmentation methods address only part of this problem. Fibular strut allografts can provide intramedullary and medial support but may be limited by graft availability, size matching, cost, and technical complexity, and they do not directly reinforce the screw–bone interface of all proximal screws. Cement augmentation can improve screw purchase, but cement alone does not create a defined intramedullary support structure or reconstruct medial column bone defects. Therefore, a composite fixation strategy is needed to simultaneously provide lateral fixation, intramedullary support, medial column stability, enhanced screw purchase, and bone defect reconstruction. To address this clinical gap, we developed the “Sandwich” technique, which combines a lateral locking plate, an intramedullary reconstruction plate, PMMA bone cement, and allogeneic cancellous bone graft to create a multidirectional support construct.

**Methods:**

Five patients with osteoporotic proximal humerus fractures and medial column defects were treated using the “Sandwich” technique. This construct consisted of a lateral locking plate, an intramedullary reconstruction plate, PMMA bone cement, and allogeneic cancellous bone graft. The lateral locking plate provided lateral fixation, whereas the intramedullary reconstruction plate provided internal structural support. PMMA bone cement was placed between the two plates to reinforce screw purchase and construct stability, and allogeneic cancellous bone graft was used to fill the residual metaphyseal and medial column defects.

**Results:**

The mean operative time was 123.0 ± 16.1 min, with an average blood loss of 206.0 ± 85.3 mL, hospital stay of 8.4 ± 1.1 days, and mean hospitalization cost of 56,258.4 ± 6,152.1 CNY. At 12 months postoperatively, all fractures achieved radiographic union without loss of reduction, screw penetration, or varus collapse. The mean CMS was 87.0 ± 6.0 and the mean Neer score was 86.0 ± 6.1. No cases of avascular necrosis or fixation failure were observed.

**Conclusion:**

The “Sandwich” technique provides strong medial and intramedullary support, improves screw fixation, and enhances overall biomechanical stability in osteoporotic proximal humerus fractures. It achieved excellent healing and functional recovery with minimal complications, representing a promising surgical alternative to traditional fibular graft-assisted fixation.

## 1 Introduction

Proximal humerus fractures (PHFs) rank as the third most common type of fracture in osteoporotic patients, with their incidence closely linked to the degree of population aging. As the global elderly population continues to expand, the prevalence of PHFs is expected to increase substantially. In cases where PHFs are accompanied by medial column disruption, the lateral locking plate combined with an intramedullary fibular segment (LLP-IFS) has become the most widely accepted and technically mature approach to enhance medial support. Both autologous and allogeneic fibular grafts have demonstrated satisfactory clinical outcomes, significantly improving the prognosis of patients with varus-type PHFs. However, several limitations remain. In osteoporotic patients, autologous fibular grafts often provide insufficient mechanical strength, and graft harvesting can cause additional trauma and donor-site morbidity. Although allogeneic fibular grafts eliminate donor-site complications, they pose risks of immune rejection, disease transmission, and infection, and their high cost may create a financial burden for patients. Moreover, osteoporotic bone is inherently fragile and prone to comminution after injury (1); further fragmentation may occur during reduction or fixation, while stress concentration at the bone–implant interface can lead to secondary fractures. Impaired callus mineralization delays healing, and poor cancellous bone quality compromises screw purchase, resulting in implant loosening or pullout (2). Consequently, osteoporotic fractures are frequently complicated by fixation failure and delayed union due to compromised bone quality (3). Although lateral locking plate fixation is widely used for displaced proximal humerus fractures, fixation failure remains an important concern in elderly osteoporotic patients, particularly when medial column defects are present. Osteoporosis leads to decreased cancellous bone quality and reduced screw purchase. Meanwhile, medial column comminution and metaphyseal bone defects weaken the ability of the proximal humerus to resist varus and axial loading. Therefore, insufficient medial support may result in loss of reduction, varus collapse, screw penetration, and fixation failure.

Existing augmentation methods can address some of these problems, but limitations remain. Fibular strut augmentation can provide intramedullary and medial structural support; however, its clinical use may be limited by graft availability, size matching, cost, and technical complexity. In addition, fibular strut augmentation mainly improves structural support and does not directly enhance the stability of the screw–bone interface for all proximal screws. Cement augmentation can improve screw purchase, but cement alone primarily acts at the screw–bone interface and cannot independently provide a defined intramedullary load-bearing structure or reconstruct medial column bone defects. Therefore, for osteoporotic proximal humerus fractures with medial column defects and metaphyseal bone voids, a composite fixation strategy is still needed to simultaneously provide lateral fixation, intramedullary support, medial column stability, screw purchase enhancement, and bone defect reconstruction. To overcome these challenges, we developed a novel “Sandwich” surgical technique, termed the lateral locking plate–intramedullary reconstruction plate with bone cement (LLP-IRPBC) method, designed to provide enhanced medial and intramedullary support, improve biomechanical stability, and optimize fixation outcomes in osteoporotic PHFs.

## 2 Methods

### 2.1 Study population

This study was designed as a retrospective case series. Five patients with osteoporotic proximal humerus fractures who were treated using the “Sandwich” technique at the Second Hospital of Jilin University between August 2022 and March 2025 were included. All patients underwent preoperative imaging and bone mineral density assessment and received regular postoperative follow-up. The study population consisted of one male and four female patients, with an age range of 53–75 years and a mean age of 66.20 ± 8.23 years. The fracture involved the left side in two patients and the right side in three patients. According to the Neer classification, there was one two-part fracture, three three-part fractures, and one four-part fracture. All cases were closed proximal humerus fractures caused by falls. No preoperative shoulder dislocation, rotator cuff injury, or other concomitant shoulder injuries that could affect surgical decision-making were identified. Preoperative DXA showed that the *T*-scores of the lumbar spine (L1–L4) and femoral neck ranged from −2.8 to −3.8, with a mean value of −3.24 ± 0.40, consistent with the clinical characteristics of osteoporotic fractures. The baseline characteristics and preoperative assessment findings are presented in Table 1. This study was approved by the Ethics Committee of the Second Hospital of Jilin University (approval number: 2024246) and was conducted in accordance with the principles of the Declaration of Helsinki. Informed consent was obtained from all individual participants included in this study.

**Table 1:** Patient characteristics and preoperative evaluation findings.

Case	Age (years)	Sex	Inju re side	Injury mechanism	Neer classification	DXA *T*-score	Preoperative x-ray/3D CT findings	Medial column defect	Shoulder dislocation	Rotator cuff injury	Relevant comorbidities
1	53	Male	Right	Fall	Two	−2.8	Closed PHF; displacement, comminution, and medial column evaluated by x-ray and 3D CT.	Present	No	No	No
2	65	Female	Left	Fall	Three	−3.0	Closed PHF; displacement, comminution, and medial column evaluated by x-ray and 3D CT.	Present	No	No	Diabetes
3	68	Female	Right	Fall	Three	−3.1	Closed PHF; displacement, comminution, and medial column evaluated by x-ray and 3D CT.	Present	No	No	Diabetes
4	70	Female	Left	Fall	Three	−3.5	Closed PHF; displacement, comminution, and medial column evaluated by x-ray and 3D CT.	Present	No	No	Diabetes; Hypertension
5	75	Female	Right	Fall	Three	−3.8	Closed PHF; displacement, comminution, and medial column evaluated by x-ray and 3D CT.	Present	No	No	Hypertension

PHF, proximal humerus fracture; DXA, dual-energy x-ray absorptiometry; CT, computed tomography. *T*-score was obtained from preoperative DXA evaluation. Bracketed fields should be replaced with patient-specific data from the medical records before submission.

Inclusion Criteria for Patients Treated with the “Sandwich” Technique. Patients were eligible for treatment with the “Sandwich” technique if they met all of the following criteria: (1) Osteoporosis diagnosed by DXA examination; (2 Proximal humerus fracture classified as Neer two-part or more complex fractures; (3) Medial column defect present in the proximal humerus fracture; (4) No severe pre-existing comorbidities or complications that would preclude surgery or interfere with postoperative shoulder rehabilitation; (5) Exclusion of pathological, old (chronic), or open fractures.

### 2.2 Preoperative preparation and relevant examinations

Prior to surgery, the affected upper limb was suspended over the chest using a triangular sling, with the elbow flexed at 90°, to minimize shoulder movement, reduce fracture displacement, and alleviate pain.

All patients underwent routine DXA examination, standard anteroposterior and lateral radiographs of the shoulder, and three-dimensional CT scans of the shoulder joint preoperatively to evaluate fracture severity and displacement (Figure 1).

**Figure 1:**
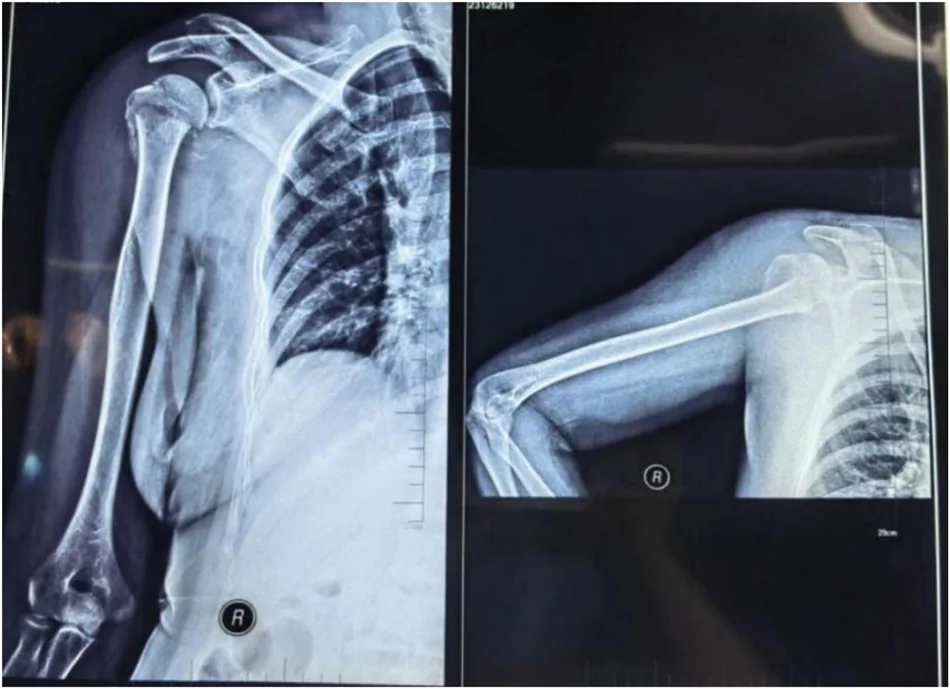
X-ray of the right proximal humerus fracture in a patient.

Comprehensive preoperative evaluations were performed, including chest CT, electrocardiogram (ECG), complete blood count, coagulation profile, immunological tests, liver and kidney function tests, electrolytes, and blood glucose levels, to rule out any surgical contraindications. The affected upper limb was suspended over the chest using a triangular sling with the elbow flexed at 90° to minimize shoulder movement, reduce fracture displacement, and alleviate pain. All patients underwent routine DXA assessment, standard anteroposterior and lateral shoulder radiographs, and three-dimensional CT scans of the shoulder joint to evaluate fracture severity and displacement (Figure 2). The surgical procedure, along with potential risks and complications, was thoroughly explained to the patients and their family members, and written informed consent was obtained. Patients were instructed to fast and abstain from drinking for 8 h before the operation, and prophylactic antibiotics were administered preoperatively.

**Figure 2:**
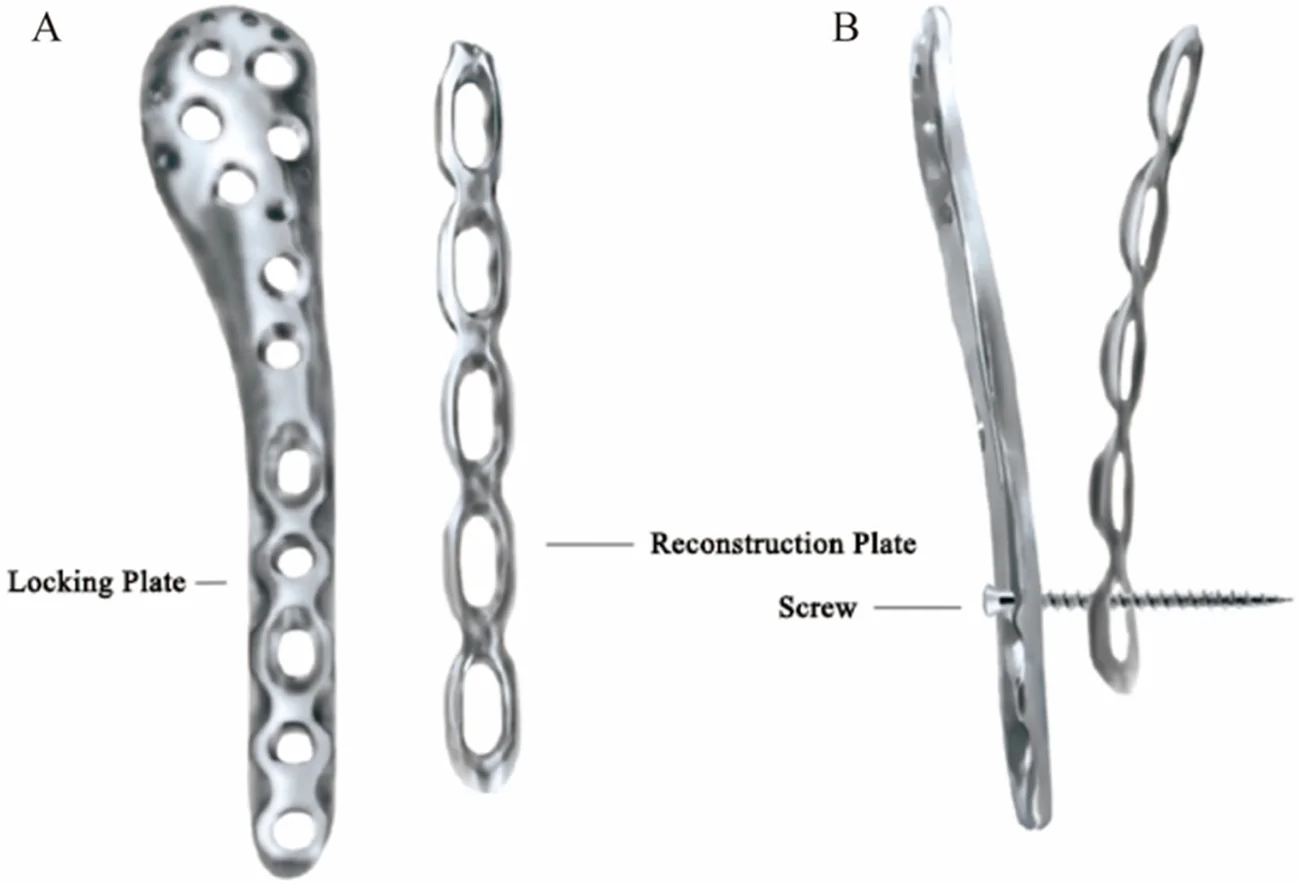
**(A)** Selection of appropriately sized plates: a lateral locking plate (90 mm) and a reconstruction plate (65 mm); **(B)** Assembly of the two plates, with the reconstruction plate shaped and pre-bent as needed.

### 2.3 Surgical technique

All surgeries were performed by a single experienced attending surgeon. Under general anesthesia, patients were placed in the beach-chair position, with the affected shoulder elevated and the head turned to the contralateral side to fully expose the operative shoulder. The operative field was disinfected with povidone-iodine solution, beginning at the planned deltopectoral incision and extending outward to include the affected shoulder, clavicular region, proximal upper arm, axillary region, lateral anterior chest wall, and scapular region. After the disinfectant had dried, sterile towels and surgical drapes were applied, while sufficient exposure of the affected shoulder and upper limb was maintained for intraoperative manipulation. According to the fracture morphology, appropriate lengths of the lateral locking plate and reconstruction plate were selected. In this study, the lateral locking plate was a proximal humeral locking plate, and the reconstruction plate was a 3.5-mm reconstruction plate. Both plates were manufactured by WEGO Orthopaedic, Shandong Weigao Orthopaedic Materials Co., Ltd., Weihai, China. Before implantation, the two plates were pre-assembled, with the reconstruction plate placed in reverse orientation, shaped, and pre-bent as needed. Locking screws were temporarily inserted to determine the optimal height, angle, and spatial relationship between the two plates.

The surgical approach was performed through the deltopectoral interval. A curved skin incision was made, and soft tissues were gently dissected layer by layer. The cephalic vein was carefully protected, and blunt dissection was performed whenever possible. Excessive periosteal stripping and unnecessary detachment of soft tissue attachments around the fracture fragments were avoided to preserve local blood supply. The deltoid muscle was retracted laterally, and the proximal exposure was extended along the long head of the biceps tendon and the intertubercular groove, which served as important anatomical landmarks. The term “anterior fracture window” refers to the pre-existing anterior fracture gap exposed through the deltopectoral approach, mainly located in the anterior metaphyseal fracture region near the intertubercular groove. No additional cortical window was intentionally created. Through this window, hematoma, intramedullary debris, and nonviable bone fragments were removed under direct visualization, while viable cancellous fragments and soft tissue attachments were preserved whenever possible.

After preliminary fracture reduction, the lateral locking plate was positioned, with the proximal end placed approximately 5–8 mm below the tip of the greater tuberosity and the medial edge located approximately 2–4 mm posterior to the intertubercular groove. Kirschner wires were used for temporary plate fixation, and intraoperative fluoroscopy was performed to confirm satisfactory fracture reduction and appropriate plate position. The reconstruction plate was then pre-contoured according to its planned spatial relationship with the lateral locking plate. Before insertion, an anti-subsidence short locking screw was placed into the middle hole of the reconstruction plate to help prevent distal migration or subsidence of the intramedullary reconstruction plate within the medullary canal. Under direct visualization, the reconstruction plate was slowly inserted retrogradely through the anterior fracture window into the humeral medullary canal. Forceful impaction was avoided. If resistance was encountered, the plate was withdrawn slightly and gently adjusted in its anterior-posterior direction or rotational orientation before further advancement. The reconstruction plate was not used as a lever against the osteoporotic metaphyseal bone, in order to reduce the risk of additional comminution or secondary fracture. The depth and alignment of the intramedullary reconstruction plate were repeatedly confirmed using Kirschner wire positioning and intraoperative fluoroscopy.

After satisfactory positioning of the intramedullary reconstruction plate, a Kirschner wire was inserted through the corresponding distal hole of the lateral locking plate and passed through or adjacent to the predetermined hole of the intramedullary reconstruction plate to temporarily maintain the depth and rotational alignment of the plate. Additional Kirschner wires were sequentially inserted through the remaining corresponding screw holes. If the intramedullary reconstruction plate obstructed the path of a proximal Kirschner wire, the plate was gently adjusted anteriorly or posteriorly using forceps to allow smooth insertion of the Kirschner wire into the humeral head without compromising the supporting function of the plate. After satisfactory alignment was confirmed by fluoroscopy, the Kirschner wires were sequentially replaced with locking screws. The tips of the proximal screws were positioned within 5 mm of the subchondral bone beneath the humeral head articular surface. Depending on the fracture pattern and plate alignment, it may not always be possible for multiple screws to pass simultaneously through both the lateral locking plate and the intramedullary reconstruction plate. In such cases, at least one screw was required to link the two plates and prevent subsidence of the intramedullary reconstruction plate within the medullary canal.

Bone grafting and cement augmentation were then performed through the anterior fracture window under direct visualization. Allogeneic cancellous bone graft was first placed into the proximal humeral head, metaphyseal region, medial defect area, and posterior fracture region, while an appropriate space was preserved between the lateral locking plate and the intramedullary reconstruction plate for bone cement placement. Polymethylmethacrylate bone cement (PMMA bone cement; PALACOS R + G, Heraeus Medical GmbH, Germany) was applied after satisfactory fracture reduction, lateral locking plate positioning, intramedullary reconstruction plate insertion, and preliminary confirmation of the spatial relationship between the two plates using Kirschner wires and locking screws. Approximately 5 mL of dough-stage PMMA bone cement was introduced through the anterior fracture window into the space between the lateral locking plate and the intramedullary reconstruction plate. The cement filled the medullary space between the two plates, reinforced screw purchase, and strengthened the plate-to-plate construct. During cement polymerization, approximately 20 mL of sterile 0.9% sodium chloride injection (Chenxin Pharmaceutical Co., Ltd., China) was used for local irrigation and cooling to reduce the risk of thermal injury caused by the exothermic reaction of PMMA bone cement. After the cement had fully cured, additional allogeneic cancellous bone graft was placed around the cement and within the residual metaphyseal and medial column defects to assist bone defect filling and medial column reconstruction. In this manner, the lateral locking plate, PMMA bone cement, intramedullary reconstruction plate, and cancellous bone graft together formed a multidirectional “Sandwich” support construct.

Passive movement of the affected shoulder was performed intraoperatively to confirm good range of motion and stable internal fixation without loosening. After final fluoroscopic confirmation of fracture reduction, implant position, screw length, and construct stability, the surgical site was irrigated, hemostasis was achieved, and a drain was placed. The subcutaneous tissue and skin were closed layer by layer, and the incision was covered with sterile dressing. Surgical time and intraoperative blood loss were recorded. The commercial information regarding implants and materials is provided only to improve methodological transparency and reproducibility and does not imply endorsement, preference, or promotion of any specific product.

Passive movement of the affected shoulder was performed intraoperatively to confirm good range of motion and stable internal fixation without loosening. After final fluoroscopic confirmation of fracture reduction, implant position, screw length, and construct stability, the surgical site was irrigated, hemostasis was achieved, and a drain was placed. The subcutaneous tissue and skin were closed layer by layer, and the incision was covered with a sterile dressing. Surgical time and intraoperative blood loss were recorded. Postoperatively, cefuroxime sodium was administered intravenously at a dose of 1.5 g every 12 h for 3 days. The mean total perioperative dose was approximately 10.5 g per patient. The commercial information regarding implants and materials is provided only to improve methodological transparency and reproducibility and does not imply endorsement, preference, or promotion of any specific product.

### 2.4 Postoperative management

The right upper limb was supported with a sling for protection. Prophylactic antibiotics were administered for the first three postoperative days to reduce the risk of infection, and the wound was disinfected and dressed every other day. On the second postoperative day, as there was no significant drainage, the surgical drain was removed, and the patient was discharged on the fourth postoperative day. Patients were encouraged to begin early functional exercises to prevent joint stiffness, starting from the wrist and elbow and gradually progressing to shoulder exercises, with the range, frequency, and intensity of movements gradually increased. Within the first four weeks, exercises were primarily passive, including pendulum movements of the shoulder; after four weeks, patients gradually transitioned to non-weight-bearing active exercises, such as shoulder flexion and elevation. Between 6–8 weeks, normal functional exercises were allowed according to the recovery of upper limb function. At 12 weeks, after radiographic confirmation of fracture healing, progressive weight-bearing exercises could be initiated. Patients were advised to maintain adequate nutrition and a balanced diet and to attend regular follow-up visits. Length of hospital stay and hospitalization costs were recorded.

## 3 Results

Patients were followed up at 1, 3, 6, and 12 months postoperatively. Standard anteroposterior and lateral radiographs of the shoulder were obtained to evaluate fracture healing, and shoulder joint function was assessed. The Constant-Murley Score (CMS) was used to evaluate pain, activities of daily living (ADL), range of motion (ROM), and muscle strength, with a total score calculated. The Neer scoring system was also applied, recording pain, function, ROM, and anatomical alignment to generate a total score. Poor shoulder functional outcomes were defined as a CMS <70 points (4) and a Neer score <70 points at the final follow-up (5). Postoperative complications were also recorded.

The mean operative time for the five patients was 123.00 ± 16.05 min, with an average intraoperative blood loss of 206.00 ± 85.32 mL. The mean hospital stay was 8.40 ± 1.14 days, and the mean hospitalization cost was 56,258.40 ± 6,152.10 CNY (Table 2).

**Table 2:** Surgical data of the patients.

Variable	Max	Min	Mean (*X¯*)	SD
Operation time (min)	145	105	123.00	16.05
Intraoperative bleeding (mL)	300	80	206.00	85.32
Hospital stay (days)	10	7	8.40	1.14
Hospitalization cost (USD)	8,912.92	6,674.17	7,813.67	854.46

The trend of shoulder function recovery over time in the five patients is shown in Figure 3. At 12 months postoperatively, the mean Constant-Murley Score (CMS) was 87.00 ± 5.96, and the mean Neer score was 86.00 ± 6.12. No cases of poor shoulder functional outcome were observed.

**Figure 3:**
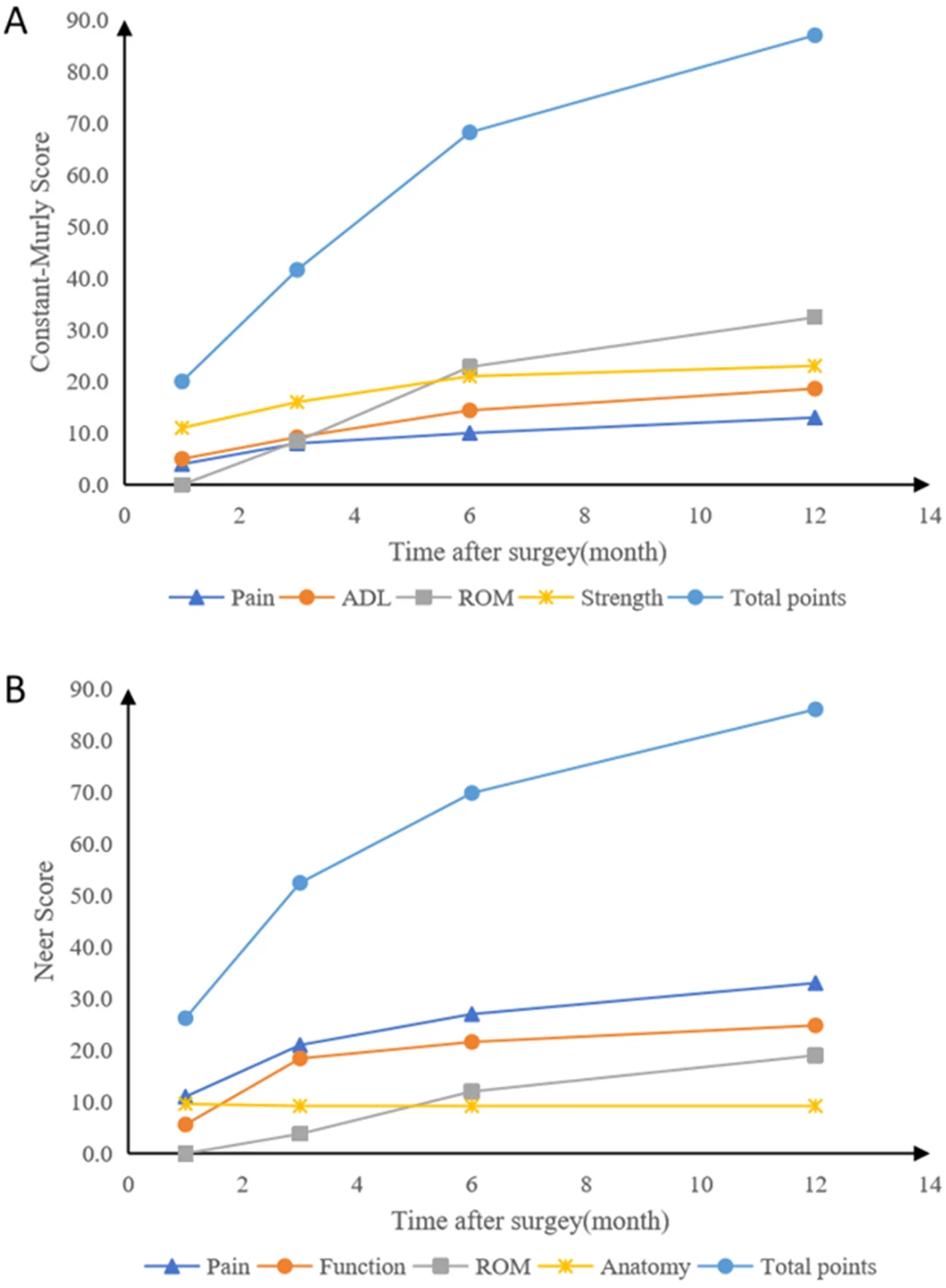
Serial changes in postoperative functional scores at 1, 3, 6, and 12 months. **(A)** Changes in the Constant–Murley total score and its component scores, including pain, activities of daily living, range of motion, and strength. **(B)** Changes in the Neer total score and its component scores, including pain, function, range of motion, and anatomy. Both total scores showed progressive improvement during the follow-up period. ADL, activities of daily living; ROM, range of motion.

Among the five patients, one had comorbid hypertension and diabetes, one had diabetes and depression, and two had hypertension. One patient with diabetes and depression developed a postoperative incision infection, which healed after regular dressing changes. Another patient with hypertension experienced cerebral infarction 4 months after surgery, presenting with numbness in the left lower limb; symptoms improved after internal medicine treatment without affecting shoulder function or follow-up. All patients achieved satisfactory fracture healing, with no cases of screw penetration, humeral head varus collapse, loss of reduction, or avascular necrosis of the humeral head observed (Figure 4).

**Figure 4:**
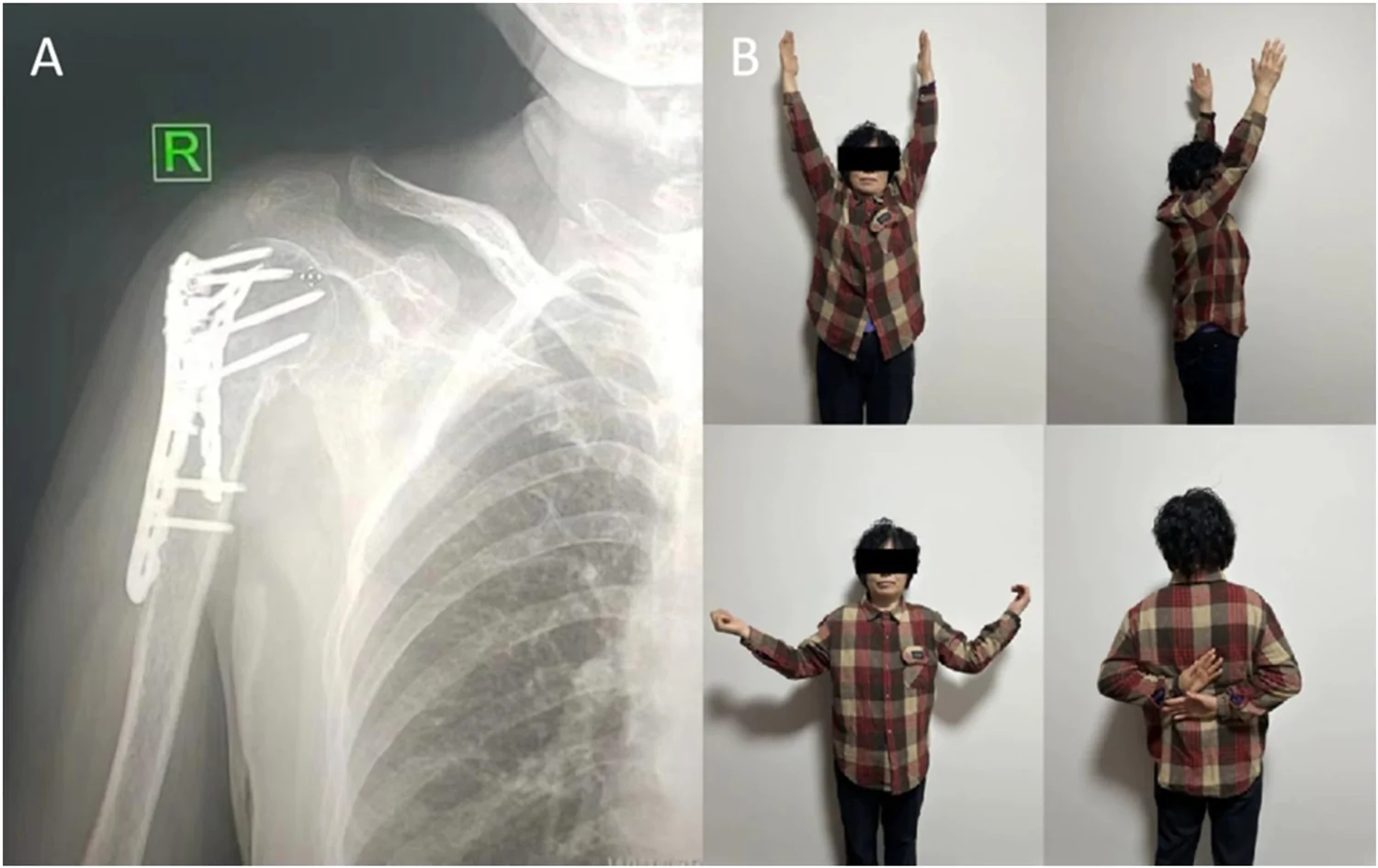
Radiographic and functional outcomes during follow-up. **(A)** Anteroposterior radiograph obtained 6 months after surgery, showing maintained fracture reduction, stable implant position, and satisfactory fracture healing. **(B)** Shoulder function at 12 months after surgery, demonstrating abduction, forward elevation, external rotation, and internal rotation.

## 4 Discussion

Osteoporosis is a systemic skeletal disease characterized by reduced bone mass and deterioration of bone microarchitecture, leading to impaired biomechanical stability of the bone (6). It primarily affects postmenopausal women but also occurs in elderly men (7). According to the World Health Organization, a decrease of one standard deviation in bone mineral density increases the relative risk of osteoporotic fractures by approximately 2.6-fold (8). Epidemiological studies have shown that the incidence of proximal humerus fractures (PHFs) significantly increases among individuals over 50 years of age, particularly in women, and these fractures are mostly the result of low-energy trauma, such as falls from standing height (9–11). The incidence ratio of PHFs between women and men is approximately 1:1 before the age of 50 but increases to 4:1 after 50 years of age, which may be attributed to factors such as a higher risk of falls in the elderly, unpredictable trauma, increased life expectancy among women, and a greater susceptibility to osteoporosis (12).

With advancing age, the relative bone mass loss in the upper limbs is approximately three times greater than that in the lower limbs, particularly among elderly women (13). However, cortical bone morphology varies substantially across different anatomical regions. Since the humerus is a non–weight-bearing bone, the proximal humerus is especially prone to rapid bone loss after menopause (14, 15). The cancellous bone of the humeral head has the highest bone mineral density (BMD) beneath the subchondral cartilage, while the area near the anatomical neck shows decreased BMD—a pattern that becomes more pronounced in osteoporosis. The site-specific reduction in cancellous bone in osteoporotic patients is most evident in the metaphyseal region, which experiences greater bone loss compared with the humeral head (16). Compared with the lateral column, the medial column of osteoporotic humeri demonstrates significantly lower BMD. Helfen et al. reported that cortical thinning and increased porosity in the surgical neck of the humerus primarily occur after the age of 65, with marked regional variations in cortical bone among postmenopausal women, especially in the metaphyseal medial column (17). Compared with the humeral head, greater tuberosity, and lesser tuberosity, the medial column exhibits the most pronounced age-related decline in bone density. Consequently, proximal humeral fractures (PHFs) in osteoporotic patients are often accompanied by medial column bone defects, rendering the region incapable of withstanding compressive forces from the humeral head and leading to postoperative fixation instability and associated complications. Gardner et al. emphasized that mechanical support from the medial column is crucial for maintaining fracture reduction in PHFs treated with locking plate fixation. Both medial comminution and insufficient medial support have been identified as independent risk factors for loss of reduction (18).

The injury mechanism of proximal humeral fractures (PHFs) varies with age and generally presents a bimodal distribution: high-energy trauma is more common in younger individuals, whereas low-energy injuries predominate in the elderly. When evaluating patients with PHFs, surgeons must obtain a thorough medical history and perform a comprehensive physical examination, paying close attention to the patient's overall functional status, such as activity level, hand dominance, and living conditions. It is also essential to assess any previous shoulder trauma or rotator cuff dysfunction in order to determine appropriate treatment options. In elderly patients, PHFs are typically considered fragility fractures, and surgical fixation may be indicated for displaced two-part, three-part, or four-part fractures of the proximal humerus.

We utilized a reconstruction plate instead of a fibular graft to restore medial support, and all patients achieved favorable postoperative outcomes. Previous studies have shown a correlation between screw stability and regional bone mineral density (BMD) in the proximal humerus; Tingart et al. reported a significant relationship between trabecular bone density and screw pullout strength, noting that low BMD often fails to provide adequate anchorage for plates and screws due to shear forces at the bone–implant interface (19). Building upon these findings, the “Sandwich” technique in this study addresses age-related osteoporotic changes, including reduced trabecular bone density in the humeral head, markedly decreased BMD in the medial column, cortical thinning, increased porosity, and weakened medial support. Bone cement was applied between the two plates to fill the gap and encapsulate the proximal screws, thereby enhancing screw fixation strength. Additionally, cancellous bone granules were used to fill bone defects caused by impaction during reduction; their porous structure provides a larger surface area for vascular and new bone ingrowth, offers strong osteoconductivity, is cost-effective, and can be produced in bulk. After defatting, decellularization, and sterilization, their immunogenicity is significantly reduced and the risk of infection is minimized (20). Thus, bone grafting in defect areas after internal fixation serves to repair bone loss, promote fracture healing, and reconstruct the medial column.

In the study by Chen et al. (21), the use of LLP-IFS for treating elderly patients with PHFs resulted in a mean operative time of 126.00 ± 48.00 min and an intraoperative blood loss of 238.00 ± 83.00 mL. In the present study, the “Sandwich” technique for osteoporotic PHFs achieved a mean operative time of 123.00 ± 16.05 min and an intraoperative blood loss of 206.00 ± 85.32 mL, which is comparable to the results reported by Chen et al. This suggests that, in terms of overall procedural complexity and technical difficulty, LLP-IFS and the “Sandwich” method may not differ substantially, and their perioperative safety profiles are likely similar. It is noteworthy that operative time and blood loss are influenced by multiple factors, including fracture type and severity, the patient's coagulation function, the surgeon's skill level, and the coordination of the surgical team, all of which collectively determine the complexity and risk of the procedure.

From the patient's perspective, one of the most important parameters is the ability to restore quality of life (QoL), as PHFs can affect QoL on multiple levels. Pain and restricted range of motion negatively impact physical functioning and may lead to loss of independence and mobility, which can, in turn, affect social engagement and contribute to emotional and psychological issues in elderly patients, further compromising overall QoL. Therefore, evaluating QoL is clinically important, as it supports patient-centered care and informs treatment decisions. Despite its significance, many studies assessing outcomes of PHFs in elderly patients report markedly reduced postoperative QoL (22). Commonly used tools for evaluating health-related QoL after PHFs include the Constant-Murley Score (CMS) and the Neer score. The CMS, developed by Pierre Constant and Stuart Murley in 1980, assesses postoperative shoulder function through four components: pain, activities of daily living (ADL), active range of motion (ROM), and muscle strength. It is internationally recognized, precise, and applicable to a variety of shoulder disorders, including fractures, rotator cuff injuries, and arthritis. The Neer score, specifically designed for functional assessment after PHFs, was proposed by Charles S. Neer in 1970 and combines clinical function with imaging results to evaluate pain, functional recovery, ROM, and anatomical reduction quality. It is often used alongside CMS to validate the efficacy of surgical techniques. In the present study, at 12 months postoperatively following the “Sandwich” fixation, the mean CMS and Neer scores were 87.00 ± 5.96 and 86.00 ± 6.12, respectively, demonstrating satisfactory functional outcomes. Comparatively, Lee and Tuerxun et al. reported CMS scores of 87.80 ± 5.60 and 83.70 ± 8.60 in elderly PHF patients treated with LLP-IFS (23, 24). These results suggest that both fixation methods achieve comparable outcomes in restoring basic shoulder function and daily activities, effectively meeting patients' functional needs and improving their QoL.

Locking plate fixation is a commonly used surgical method for treating osteoporotic proximal humeral fractures (PHFs), especially for displaced fractures involving two or more parts according to Neer classification. Despite providing good angular stability and anatomical conformity, the complication rate remains relatively high. Screw penetration is the most frequent complication, with an incidence ranging from 0% to 23% (25), and can be categorized as primary or secondary. Primary screw penetration occurs intraoperatively as an iatrogenic injury, for which routine use of three-plane fluoroscopy is recommended to reduce its occurrence. Secondary screw penetration is often caused by osteoporosis, insufficient medial support, or overly long screws; Gardner et al. found that loss of medial support increases the rate of screw penetration by fivefold (18). Varus collapse is another common postoperative complication, occurring in 16.3% of cases, often limiting shoulder range of motion, particularly during forward elevation and abduction. Insufficient medial support may contribute to varus collapse, and patients with initial varus displacement have a higher risk of varus malunion postoperatively (40.4%) (9). Loss of reduction occurs in 4.2%–13.7% of locking plate-treated PHFs and can reach up to 40% in complex fractures, such as Neer three- and four-part fractures (26). Reduced bone density weakens screw anchorage, and factors such as medial support deficiency, poor reduction, or improper screw placement can disrupt biomechanical balance, accelerating loss of reduction (27). Subacromial impingement has an incidence of approximately 4.8%, mainly due to malpositioned lateral plates, malunion of the greater tuberosity, or secondary varus collapse of the humeral head (28). Proper plate positioning and stable medial support are essential to reduce this complication. Full-arc passive shoulder motion before wound closure is critical for assessing impingement. Ricchetti et al. suggested that positioning the plate 15–20 mm below the tip of the greater tuberosity may help prevent impingement (29). Avascular necrosis of the humeral head is the most severe complication following locking plate fixation, with an incidence of 4.4%–10.8% (30). Impaired blood supply is the primary cause, and the integrity of the medial soft tissue, the length of the posteromedial metaphyseal column, and fracture type are key predictors of humeral head avascular necrosis. Fjalestad et al. noted that in displaced three- and four-part PHFs, the incidence of humeral head avascular necrosis is higher with nonoperative treatment compared to surgical fixation (31).

Multiple studies have confirmed that augmenting medial support can significantly reduce the incidence of complications. Nie et al. reported a complication rate of 21.8% for PHFs treated with locking plates alone, compared with 8.9% for those treated with LLP-IFS (32). Similarly, Cui et al. found complication rates of 28.57% and 12.0% for locking plate fixation alone vs. LLP-IFS, respectively (33). In the present study, all five PHF patients treated with the “Sandwich” technique achieved satisfactory fracture healing and significant improvement in shoulder function, with no related complications observed. The clinical efficacy was clear and satisfactory; however, further studies with larger sample sizes are needed to evaluate the long-term outcomes and safety of the “Sandwich” method.

The clinical application of the “Sandwich” technique demonstrates its effectiveness in treating osteoporotic PHFs and provides a clear direction for clinical management. The reconstruction plate provides direct load-bearing support beneath the humeral head articular surface while also supporting the medial column, enhancing biomechanical stability after fracture fixation. Augmentation with bone cement effectively prevents screw loosening and implant failure, and adequate bone grafting promotes fracture healing and medial column reconstruction. Following proper fracture healing and shoulder function recovery, the final surgical outcomes were satisfactory, with no postoperative complications, including screw penetration, implant loosening, varus displacement, or humeral head avascular necrosis. Compared with autologous or allogeneic fibular grafts, the “Sandwich” technique offers several advantages: first, it provides robust biomechanical stability, ensuring strong medial support and significantly reducing micro-movement and subsequent bone resorption; second, it markedly enhances screw fixation strength, effectively preventing screw loosening and implant failure; third, it reduces the risk of infection or disease transmission; fourth, intramedullary placement of the reconstruction plate with medial cortical bone grafting facilitates the restoration of normal medial column morphology after fracture healing; fifth, the overall structural stability allows for early functional exercises, minimizing muscle atrophy, tissue adhesion, and restricted mobility. Adequate bone grafting at the fracture site further promotes healing and recovery, reducing the incidence of postoperative complications and the need for revision surgery.

In conclusion, the “Sandwich” technique is suitable for two-part, three-part, and four-part proximal humeral fractures with displacement of the humeral head or greater tuberosity. For minimally displaced fractures (<1 cm), conservative treatment remains the first choice. In cases of severely comminuted proximal humeral fractures, humeral head split fractures, fracture-dislocations, high risk of internal fixation failure, or an elevated likelihood of humeral head necrosis, shoulder arthroplasty should be considered. Given the presence of comorbidities, revision surgery is often not feasible for many elderly patients; therefore, surgeons must carefully evaluate all relevant factors and tailor the surgical plan to each patient's individual needs.

However, these potential advantages are currently based mainly on the technical design rationale and early clinical observations, and they still require further validation through biomechanical testing and prospective clinical studies. This study has several limitations. First, this was an initial case series of a novel fixation technique, and only five patients were included. The small sample size inevitably limited the statistical power and clinical strength of the findings and reduced the generalizability of the results to a broader patient population. Therefore, reliable subgroup analysis, risk-factor analysis, or statistical comparison could not be performed. The present findings should be interpreted as preliminary clinical experience with the “Sandwich” technique in carefully selected patients rather than definitive evidence of clinical effectiveness.

Second, the study population included a higher proportion of female patients, which may have influenced the interpretation of the results. Postmenopausal women are commonly associated with decreased bone mineral density, poorer cancellous bone quality, weakened medial column support, and reduced screw purchase. These factors may affect the maintenance of fracture reduction, fixation stability, and postoperative functional recovery. However, the predominance of female patients is also consistent with the epidemiological characteristics of osteoporotic proximal humerus fractures, as these fractures occur more frequently in elderly women. Therefore, the present findings should be more appropriately interpreted as preliminary clinical experience with this technique in a patient population mainly consisting of elderly women with osteoporotic fractures. Because of the limited sample size, this study was unable to further compare radiographic healing, complication rates, or functional recovery between male and female patients.

Third, no control group was included in this study, which limits our ability to evaluate the true clinical benefit of this technique. At present, the “Sandwich” technique cannot be directly compared with locking plate fixation alone, fibular strut augmentation, cement augmentation alone, or other medial column reconstruction strategies. Therefore, this study cannot demonstrate that this technique is superior to existing fixation methods. Although all patients in this series achieved fracture healing, and no major complications such as screw penetration, loss of reduction, varus collapse, fixation failure, or avascular necrosis of the humeral head were observed during follow-up, these findings should still be regarded as preliminary observations from an early case series. Future prospective studies with larger sample sizes, a more balanced sex distribution, appropriate control groups, and longer follow-up are required to further validate the safety, effectiveness, indications, and long-term outcomes of this technique.

### 4.1 Limitations

The “Sandwich” fixation technique proposed in this study provides a novel approach for the treatment of proximal humeral fractures, but it still has certain limitations. First, although the biomechanical stability of this method is theoretically superior to that of single plating or cement augmentation alone, large-scale prospective clinical studies are lacking, and its long-term outcomes have not been fully validated. Second, the use of bone cement around the joint carries potential risks, including thermal injury, cement leakage, and impacts on local blood supply, which require particular caution in elderly osteoporotic patients. Third, the “Sandwich” fixation procedure is relatively complex, demanding high surgical skill, and the increased number of implants may expand the range of soft tissue dissection, thereby increasing the risk of infection and compromised blood supply. Finally, this study did not fully evaluate the applicability of this technique across different fracture types and age groups; multicenter randomized controlled trials are still needed to confirm its safety and clinical advantages.

## 5 Conclusion

The “Sandwich” technique represents a promising surgical option for treating osteoporotic proximal humeral fractures (PHFs). Postoperatively, patients achieved satisfactory fracture healing and significant improvement in shoulder function, with no observed complications such as varus collapse, loss of reduction, or avascular necrosis of the humeral head, resulting in overall favorable clinical outcomes.

## Data Availability

The original contributions presented in the study are included in the article/Supplementary Material, further inquiries can be directed to the corresponding author.
