# Supplementary material for: The “Sandwich” technique using a lateral locking plate and intramedullary reconstruction plate with bone cement for osteoporotic proximal humerus fractures: a case series
Source: Front Surg. 2026 Oct 7;13:1745435. doi: 10.3389/fsurg.2026.1745435 (PMC13683606; doi:10.3389/fsurg.2026.1745435)
Supplement: Supplementary file 1 [file Datasheet1.docx]

Supplementary Material

# 1 Supplementary Data

**Table 1. Patient characteristics and preoperative evaluation findings**

| **Case** | **Age (years)** | **Sex** | **Inju re side** | **Injury mechanism** | **Neer classification** | **DXA T-score** | **Preoperative X-ray/3D CT findings** | **Medial column defect** | **Shoulder dislocation** | **Rotator cuff injury** | **Relevant comorbidities** |
| --- | --- | --- | --- | --- | --- | --- | --- | --- | --- | --- | --- |
| 1 | 53 | Male | Right | Fall | Two | −2.8 | Closed PHF; displacement, comminution, and medial column evaluated by X-ray and 3D CT. | Present | No | No | No |
| 2 | 65 | Female | Left | Fall | Three | −3.0 | Closed PHF; displacement, comminution, and medial column evaluated by X-ray and 3D CT. | Present | No | No | Diabetes |
| 3 | 68 | Female | Right | Fall | Three | −3.1 | Closed PHF; displacement, comminution, and medial column evaluated by X-ray and 3D CT. | Present | No | No | Diabetes |
| 4 | 70 | Female | Left | Fall | Three | −3.5 | Closed PHF; displacement, comminution, and medial column evaluated by X-ray and 3D CT. | Present | No | No | Diabetes; Hypertension |
| 5 | 75 | Female | Right | Fall | Three | −3.8 | Closed PHF; displacement, comminution, and medial column evaluated by X-ray and 3D CT. | Present | No | No | Hypertension |

**Abbreviations:** PHF, proximal humerus fracture; DXA, dual-energy X-ray absorptiometry; CT, computed tomography. T-score was obtained from preoperative DXA evaluation. Bracketed fields should be replaced with patient-specific data from the medical records before submission.

Table 2. Surgical data of the patients

| Variable | Max | Min | Mean (X̄) | SD |
| --- | --- | --- | --- | --- |
| Operation time (min) | 145 | 105 | 123.00 | 16.05 |
| Intraoperative bleeding (ml) | 300 | 80 | 206.00 | 85.32 |
| Hospital stay (days) | 10 | 7 | 8.40 | 1.14 |
| Hospitalization cost (USD) | 8,912.92 | 6,674.17 | 7,813.67 | 854.46 |

# 2 Supplementary Figures

**Figure 1.** X-ray of the right proximal humerus fracture in a patient

**Figure 2.** A .Selection of appropriately sized plates: a lateral locking plate (90 mm) and a reconstruction plate (65 mm); B. Assembly of the two plates, with the reconstruction plate shaped and pre-bent as needed.

**Fig****ure 3.** A. Schematic illustration; B. Intraoperative X-ray

- **Figure 4.** Key steps of intramedullary reconstruction plate positioning and temporary Kirschner wire alignment. **A.** After placement of an anti-subsidence short screw into the middle hole of the reconstruction plate, the pre-contoured reconstruction plate was inserted retrogradely through the anterior fracture window into the humeral medullary canal. **B.** A Kirschner wire was inserted through the second distal hole of the lateral locking plate and passed precisely through the most distal hole of the intramedullary reconstruction plate, thereby confirming the planned spatial relationship between the two plates. **C.** Additional Kirschner wires were sequentially inserted through the remaining corresponding screw holes to temporarily maintain the depth and rotational alignment of the intramedullary reconstruction plate. **D.**After satisfactory alignment was confirmed, the Kirschner wires were sequentially replaced with locking screws to connect the lateral locking plate and the intramedullary reconstruction plate.

- **Figure 5.** Intraoperative fluoroscopic confirmation of the dual-plate intramedullary support construct. **A.** After fracture reduction, the lateral locking plate and the pre-contoured intramedullary reconstruction plate were positioned. Kirschner wires were used for temporary alignment and fluoroscopic confirmation of the spatial relationship between the two plates. **B.** After insertion of the proximal locking screws, the intramedullary reconstruction plate was maintained within the humeral medullary canal, forming an internal support structure beneath the humeral head. The lateral locking plate and intramedullary reconstruction plate together formed the dual-plate support framework before cement augmentation and bone grafting.

**Figure 6.** Schematic illustration of the completed multidirectional “Sandwich” construct. **A.** Aneroposterior view. **B.** Lateral view. Green indicates PMMA bone cement, and white indicates allogeneic cancellous bone graft. The construct consists of a lateral locking plate, an intramedullary reconstruction plate, PMMA bone cement, and allogeneic cancellous bone graft. The lateral locking plate provides lateral fixation, the intramedullary reconstruction plate provides intramedullary support, PMMA bone cement reinforces the screw–bone interface and plate-to-plate stability, and allogeneic cancellous bone graft fills the residual metaphyseal and medial column defects. These components together create a multidirectional support structure for osteoporotic proximal humerus fractures with medial column defects.

**Figure 7.** Serial changes in postoperative functional scores at 1, 3, 6, and 12 months. **A.** Changes in the Constant–Murley total score and its component scores, including pain, activities of daily living, range of motion, and strength. **B.** Changes in the Neer total score and its component scores, including pain, function, range of motion, and anatomy. Both total scores showed progressive improvement during the follow-up period. ADL, activities of daily living; ROM, range of motion

**Figure8.** Radiographic and functional outcomes during follow-up. **A.** Anteroposterior radiograph obtained 6 months after surgery, showing maintained fracture reduction, stable implant position, and satisfactory fracture healing. **B.** Shoulder function at 12 months after surgery, demonstrating abduction, forward elevation, external rotation, and internal rotation.
